# Supplementary material for: The Systems Biology Research Tool: evolvable open-source software
Source: BMC Syst Biol. 2008 Jun 29;2:55. doi: 10.1186/1752-0509-2-55 (PMC2446383; doi:10.1186/1752-0509-2-55)
Supplement: Additional file 1 — SBRT Archive. An archive of the current version of the Systems Biology Research Tool. [file 1752-0509-2-55-S1.zip › sbrt-1.4.0/doc/developers_guide/api/index-files/index-18.html]

 
 
 
 
 
 
R-Index
 


 

 
function windowTitle()
{
    parent.document.title="R-Index";
}
 
 
 

 

 


 
   
  
 
 
 
   
 
   
          Overview     
        Package   
        Class   
        Use   
          Tree     
          Deprecated     
       Index    
          Help     
   
 
 
  
 
 
 

 
  
   PREV LETTER   
   NEXT LETTER    
  
    FRAMES     
   NO FRAMES     
  
    All Classes  ');
  }
  //-->
 
 
    All Classes  
 


  
 
 
  
 

 A   B   C   D   E   F   G   H   I   J   K   L   M   N   O   P   Q   R   S   T   U   V   W   X   Y   Z   
    
 R  
 
   R   - Class in  sbrt.ext.R  This class is used to access the R engine safely.   RandomConstraintsGeneratorV1   - Class in  sbrt.kernel.fba.proc  This class is used to generate random flux constraints.   RandomConstraintsGeneratorV1(IrrevRxnFluxome)   - 
Constructor for class sbrt.kernel.fba.proc. RandomConstraintsGeneratorV1 
 Constructs a new random constraint generator.
   RandomConstraintsManager   - Class in  sbrt.shell.mng.fba  This class is used to manage random constraints generators.   RandomConstraintsManager()   - 
Constructor for class sbrt.shell.mng.fba. RandomConstraintsManager 
  
   RandomEnvGeneratorV1   - Class in  sbrt.kernel.fba.proc  This class is used to generate random environments for use in flux balance
 analysis.   RandomEnvGeneratorV1(IrrevRxnFluxome)   - 
Constructor for class sbrt.kernel.fba.proc. RandomEnvGeneratorV1 
 Constructs a new random environment generator.
   RandomEnvManager   - Class in  sbrt.shell.mng.fba  This class is used to manage random environment generators.   RandomEnvManager()   - 
Constructor for class sbrt.shell.mng.fba. RandomEnvManager 
  
   RandomFluxVectorManager   - Class in  sbrt.shell.mng.fba  This abstract class provides a foundation for random flux vector
 managers.   RandomFluxVectorManager()   - 
Constructor for class sbrt.shell.mng.fba. RandomFluxVectorManager 
  
   RandomObjectiveGenerator   - Class in  sbrt.kernel.fba.proc  This class is used to generate random objective functions.   RandomObjectiveGenerator(Fluxome)   - 
Constructor for class sbrt.kernel.fba.proc. RandomObjectiveGenerator 
 Constructs a new random objective function generator.
   RandomObjectiveManager   - Class in  sbrt.shell.mng.fba  This class is used to manage random objective function generators.   RandomObjectiveManager()   - 
Constructor for class sbrt.shell.mng.fba. RandomObjectiveManager 
  
   rangesEqualZero(Map<?, Interval>, double)   - 
Method in class sbrt.kernel.fba.analysis. FluxIntervalAnalyzer 
 Returns the number of provided intervals whose ranges equal zero.
   rangesEqualZero(Collection<Interval>, double)   - 
Method in class sbrt.kernel.fba.analysis. FluxIntervalAnalyzer 
 Returns the number of provided intervals whose ranges equal zero.
   rangesGreaterThanZero(Map<?, Interval>, double)   - 
Method in class sbrt.kernel.fba.analysis. FluxIntervalAnalyzer 
 Returns the number of provided intervals whose ranges are greater than zero.
   rangesGreaterThanZero(Collection<Interval>, double)   - 
Method in class sbrt.kernel.fba.analysis. FluxIntervalAnalyzer 
 Returns the number of provided intervals whose ranges are greater than zero.
   rBusy(Rengine, int)   - 
Method in class sbrt.ext.R. DummyConsole 
 Does nothing.
   rChooseFile(Rengine, int)   - 
Method in class sbrt.ext.R. DummyConsole 
 Does nothing.
   RCorrelator  < V > - Class in  sbrt.ext.R.stat  This class computes correlation statistics using  R .   RCorrelator()   - 
Constructor for class sbrt.ext.R.stat. RCorrelator 
  
   RCorrelator.Result   - Class in  sbrt.ext.R.stat  This class is used to store the results of computing a correlation using
 R.   RedundantCycleRemoval   - Class in  sbrt.shell.proc.graph_theory  This class is used   RedundantCycleRemoval(String)   - 
Constructor for class sbrt.shell.proc.graph_theory. RedundantCycleRemoval 
 Constructs a new SBML file reader.
   redundantRxns()   - 
Method in interface sbrt.kernel.fba. Fluxome 
 Returns the total number of redundant reactions contained
 in this fluxome.
   redundantRxns()   - 
Method in class sbrt.kernel.fba. IrrevRxnFluxomeV1 
 Returns the total number of redundant reactions contained in this
 fluxome.
   remove(Object)   - 
Method in class sbrt.kernel.util. UnmodifiableCollection 
 See Collection.remove(Object).
   remove()   - 
Method in class sbrt.kernel.util. UnmodifiableIterator 
 Throws  UnsupportedOperationException .
   remove(Object)   - 
Method in class sbrt.kernel.util. UnmodifiableList 
 See List.remove(Object).
   remove(int)   - 
Method in class sbrt.kernel.util. UnmodifiableList 
 See List.remove(int).
   remove(Object)   - 
Method in class sbrt.kernel.util. UnmodifiableMap 
 See Map.remove(Object).
   remove(Object)   - 
Method in class sbrt.kernel.util. UnmodifiableSet 
 See Set.remove(Object).
   remove()   - 
Method in class sbrt.shell.io. ComiFileSource 
 Throws  UnsupportedOperationException .
   remove()   - 
Method in class sbrt.shell.io. ComoFileSource 
 Throws  UnsupportedOperationException .
   remove()   - 
Method in class sbrt.shell.io.fba. CofoofSource 
 Throws  UnsupportedOperationException .
   remove()   - 
Method in class sbrt.shell.io.fba. RofoofSource 
 Throws  UnsupportedOperationException .
   remove()   - 
Method in class sbrt.shell.io.fba. SfoofSource 
 Throws  UnsupportedOperationException .
   remove()   - 
Method in class sbrt.shell.io. RomiFileSource 
 Throws  UnsupportedOperationException .
   remove()   - 
Method in class sbrt.shell.io. RomoFileSource 
 Throws  UnsupportedOperationException .
   remove()   - 
Method in class sbrt.shell.io. SerializedFileIterator 
 Throws UnsupportedOperationException.
   remove()   - 
Method in class sbrt.shell.io. SmoFileSource 
 Throws  UnsupportedOperationException .
   remove()   - 
Method in class sbrt.shell.io. TextFileLineIterator 
 Throws UnsupportedOperationException.
   REMOVE_REDUNDANT_REACTIONS   - 
Static variable in class sbrt.shell.mng.fba. NetworkInfoManager 
 The keyword used to denote whether or not redundant reactions should be
 removed.
   removeAll(Collection<?>)   - 
Method in class sbrt.kernel.util. UnmodifiableCollection 
 See Collection.removeAll(Collection).
   removeAll(Collection<?>)   - 
Method in class sbrt.kernel.util. UnmodifiableList 
 See List.removeAll(Collection).
   removeAll(Collection<?>)   - 
Method in class sbrt.kernel.util. UnmodifiableSet 
 See Set.removeAll(Collection).
   removeColumn(Object)   - 
Method in class sbrt.kernel.math.algebra. HashMatrix 
 Removes the column corresponding to the provided 
 column index.
   removeColumn(Object)   - 
Method in interface sbrt.kernel.math.algebra. MatrixBuilder 
 Removes the column corresponding to the provided 
 column index.
   removeColumn(Object)   - 
Method in class sbrt.kernel.math.algebra. RowOrTreeMatrix 
 Removes the column corresponding to the provided 
 column index.
   removeColumn(Object)   - 
Method in class sbrt.kernel.math.algebra. TreeMatrix 
 Removes the column corresponding to the provided 
 column index.
   removeRow(Object)   - 
Method in class sbrt.kernel.math.algebra. HashMatrix 
 Removes the row corresponding to the provided 
 row index.
   removeRow(Object)   - 
Method in interface sbrt.kernel.math.algebra. MatrixBuilder 
 Removes the row corresponding to the provided 
 row index.
   removeRow(Object)   - 
Method in class sbrt.kernel.math.algebra. RowOrTreeMatrix 
 Removes the row corresponding to the provided 
 row index.
   removeRow(Object)   - 
Method in class sbrt.kernel.math.algebra. TreeMatrix 
 Removes the row corresponding to the provided 
 row index.
   replaceConstraints(FluxConstraints, double)   - 
Method in interface sbrt.kernel.fba. CatalyzedFluxome 
 Returns a copy of this fluxome, but with all constraints
 replaced by those provided.
   replaceConstraints(FluxConstraints, double)   - 
Method in interface sbrt.kernel.fba. Fluxome 
 Returns a copy of this fluxome, but with all constraints
 replaced by those provided.
   replaceConstraints(FluxConstraints, double)   - 
Method in interface sbrt.kernel.fba. IrrevRxnFluxome 
 Returns a copy of this fluxome, but with all constraints
 replaced by those provided.
   replaceConstraints(FluxConstraints, double)   - 
Method in class sbrt.kernel.fba. IrrevRxnFluxomeV1 
 Returns a copy of this fluxome, but with all constraints
 replaced by those provided.
   requiredKeywords   - 
Static variable in class sbrt.shell.mng. AbstractProcessManager 
 The set of keywords required to be present in the  ManagerInput 
 objects supplied to instances of this class.
   requiredKeywords   - 
Static variable in class sbrt.shell.mng.algebra. LinearCombConverterManager 
 The set of keywords required by this process manager.
   requiredKeywords   - 
Static variable in class sbrt.shell.mng.algebra. LinearSolverManager 
 The set of keywords required by this process manager.
   requiredKeywords   - 
Static variable in class sbrt.shell.mng.algebra. MatrixConverterManager 
 The set of keywords required by this process manager.
   requiredKeywords   - 
Static variable in class sbrt.shell.mng.algebra. MultipleVectorsConverterManager 
 The set of keywords required by this process manager.
   requiredKeywords   - 
Static variable in class sbrt.shell.mng.algebra. SingleVectorConverterManager 
 The set of keywords required by this process manager.
   requiredKeywords   - 
Static variable in class sbrt.shell.mng.comb. SingleElementUnionManager 
 The set of keywords required by this process manager.
   requiredKeywords   - 
Static variable in class sbrt.shell.mng.comb. StrictSingleElementUnionManager 
 The set of keywords required by this process manager.
   requiredKeywords   - 
Static variable in class sbrt.shell.mng.fba. BiggSbmlReaderManager 
 The set of keywords required by this process manager.
   requiredKeywords   - 
Static variable in class sbrt.shell.mng.fba. BiggSbmlTranslaterManager 
 The set of keywords required by this process manager.
   requiredKeywords   - 
Static variable in class sbrt.shell.mng.fba. CatalystDeletionManager 
 The set of keywords required to be present in the  ManagerInput 
 objects supplied to instances of this class.
   requiredKeywords   - 
Static variable in class sbrt.shell.mng.fba. CdHarFluxVectorManager 
 The set of keywords required to be present in the  ManagerInput 
 objects supplied to instances of this class.
   requiredKeywords   - 
Static variable in class sbrt.shell.mng.fba. CvCatalystDeletionManager 
 The set of keywords required to be present in the  ManagerInput 
 objects supplied to instances of this class.
   requiredKeywords   - 
Static variable in class sbrt.shell.mng.fba. CvFbaOptManager 
 The set of keywords required to be present in the  ManagerInput 
 objects supplied to instances of this class.
   requiredKeywords   - 
Static variable in class sbrt.shell.mng.fba. CvMsFluxIntervalManager 
 The set of keywords required to be present in the  ManagerInput 
 objects supplied to instances of this class.
   requiredKeywords   - 
Static variable in class sbrt.shell.mng.fba. CvObjectiveFunctionManager 
 The set of keywords required to be present in the  ManagerInput 
 objects supplied to instances of this class.
   requiredKeywords   - 
Static variable in class sbrt.shell.mng.fba. CvRevRxnFluxIntervalManager 
 The set of keywords required to be present in the  ManagerInput 
 objects supplied to instances of this class.
   requiredKeywords   - 
Static variable in class sbrt.shell.mng.fba. CvRxnDeletionManager 
 The set of keywords required to be present in the  ManagerInput 
 objects supplied to instances of this class.
   requiredKeywords   - 
Static variable in class sbrt.shell.mng.fba. CvSimpleFluxIntervalManager 
 The set of keywords required to be present in the  ManagerInput 
 objects supplied to instances of this class.
   requiredKeywords   - 
Static variable in class sbrt.shell.mng.fba. ExtremeCurrentManager 
 The set of keywords required to be present in the  ManagerInput 
 objects supplied to instances of this class.
   requiredKeywords   - 
Static variable in class sbrt.shell.mng.fba. FbaManager 
 The set of keywords required to be present in the  ManagerInput 
 objects supplied to instances of this class.
   requiredKeywords   - 
Static variable in class sbrt.shell.mng.fba. FbaOptManager 
 The set of keywords required to be present in the  ManagerInput 
 objects supplied to instances of this class.
   requiredKeywords   - 
Static variable in class sbrt.shell.mng.fba. FbaSolverManager 
 The set of keywords required to be present in the  ManagerInput 
 objects supplied to instances of this class.
   requiredKeywords   - 
Static variable in class sbrt.shell.mng.fba. FluxActivityManager 
 The set of keywords required to be present in the  ManagerInput 
 objects supplied to instances of this class.
   requiredKeywords   - 
Static variable in class sbrt.shell.mng.fba. FluxCapManager 
 The set of keywords required to be present in the  ManagerInput 
 objects supplied to instances of this class.
   requiredKeywords   - 
Static variable in class sbrt.shell.mng.fba. FluxPlasticityManager 
 The set of keywords required to be present in the  ManagerInput 
 objects supplied to instances of this class.
   requiredKeywords   - 
Static variable in class sbrt.shell.mng.fba. InitialPointManager 
 The set of keywords required to be present in the  ManagerInput 
 objects supplied to instances of this class.
   requiredKeywords   - 
Static variable in class sbrt.shell.mng.fba. MetatoolFileWriterManager 
 The set of keywords required by this process manager.
   requiredKeywords   - 
Static variable in class sbrt.shell.mng.fba. MmaNotebookManager 
 The set of keywords required to be present in the  ManagerInput 
 objects supplied to instances of this class.
   requiredKeywords   - 
Static variable in class sbrt.shell.mng.fba. MsFluxIntervalManager 
 The set of keywords required to be present in the  ManagerInput 
 objects supplied to instances of this class.
   requiredKeywords   - 
Static variable in class sbrt.shell.mng.fba. MsFluxomeReducerManager 
 The set of keywords required to be present in the  ManagerInput 
 objects supplied to instances of this class.
   requiredKeywords   - 
Static variable in class sbrt.shell.mng.fba. NetworkInfoManager 
 The set of keywords required to be present in the  ManagerInput 
 objects supplied to instances of this class.
   requiredKeywords   - 
Static variable in class sbrt.shell.mng.fba. ObjectiveFunctionManager 
 The set of keywords required to be present in the  ManagerInput 
 objects supplied to instances of this class.
   requiredKeywords   - 
Static variable in class sbrt.shell.mng.fba. PalssonSbmlReaderManager 
 The set of keywords required by this process manager.
   requiredKeywords   - 
Static variable in class sbrt.shell.mng.fba. PalssonSbmlTranslaterManager 
 The set of keywords required by this process manager.
   requiredKeywords   - 
Static variable in class sbrt.shell.mng.fba. RandomConstraintsManager 
 The set of keywords required to be present in the  ManagerInput 
 objects supplied to instances of this class.
   requiredKeywords   - 
Static variable in class sbrt.shell.mng.fba. RandomEnvManager 
 The set of keywords required to be present in the  ManagerInput 
 objects supplied to instances of this class.
   requiredKeywords   - 
Static variable in class sbrt.shell.mng.fba. RandomObjectiveManager 
 The set of keywords required to be present in the  ManagerInput 
 objects supplied to instances of this class.
   requiredKeywords   - 
Static variable in class sbrt.shell.mng.fba. ReverseRxnFluxIntervalManager 
 The set of keywords required to be present in the  ManagerInput 
 objects supplied to instances of this class.
   requiredKeywords   - 
Static variable in class sbrt.shell.mng.fba. RxnDeletionManager 
 The set of keywords required to be present in the  ManagerInput 
 objects supplied to instances of this class.
   requiredKeywords   - 
Static variable in class sbrt.shell.mng.fba. RxnRemovalManager 
 The set of keywords required to be present in the  ManagerInput 
 objects supplied to instances of this class.
   requiredKeywords   - 
Static variable in class sbrt.shell.mng.fba. SbmlWriterManager 
 The set of keywords required by this process manager.
   requiredKeywords   - 
Static variable in class sbrt.shell.mng.fba. SimpleFluxIntervalManager 
 The set of keywords required to be present in the  ManagerInput 
 objects supplied to instances of this class.
   requiredKeywords   - 
Static variable in class sbrt.shell.mng.fba. SimpleRxnFileTransManager 
 The set of keywords required by this process manager.
   requiredKeywords   - 
Static variable in class sbrt.shell.mng.fba. SlpCycleIdManager 
 The set of keywords required to be present in the  ManagerInput 
 objects supplied to instances of this class.
   requiredKeywords   - 
Static variable in class sbrt.shell.mng.fba. StoichVariationManager 
 The set of keywords required to be present in the  ManagerInput 
 objects supplied to instances of this class.
   requiredKeywords   - 
Static variable in class sbrt.shell.mng.fba. SvCdHarFluxVectorManager 
 The set of keywords required to be present in the  ManagerInput 
 objects supplied to instances of this class.
   requiredKeywords   - 
Static variable in class sbrt.shell.mng.fba. SvInitialPointManager 
 The set of keywords required to be present in the  ManagerInput 
 objects supplied to instances of this class.
   requiredKeywords   - 
Static variable in class sbrt.shell.mng.fba. WwFluxomeReducerManager 
 The set of keywords required to be present in the  ManagerInput 
 objects supplied to instances of this class.
   requiredKeywords   - 
Static variable in class sbrt.shell.mng.geom. CdHarManager 
 The set of keywords required by this process manager.
   requiredKeywords   - 
Static variable in class sbrt.shell.mng.graph_theory. CycleIdManager 
 The set of keywords required to be present in the  ManagerInput 
 objects supplied to instances of this class.
   requiredKeywords   - 
Static variable in class sbrt.shell.mng.graph_theory. PathIdManager 
 The set of keywords required to be present in the  ManagerInput 
 objects supplied to instances of this class.
   requiredKeywords   - 
Static variable in class sbrt.shell.mng.graph_theory. UniqueCycleIdManager 
 The set of keywords required to be present in the  ManagerInput 
 objects supplied to instances of this class.
   requiredKeywords   - 
Static variable in class sbrt.shell.mng.stat. CorrelationManager 
 The set of keywords required by this process manager.
   requiredKeywords   - 
Static variable in class sbrt.shell.mng.stat. KendallTauManager 
 The set of keywords required by this process manager.
   requiredKeywords   - 
Static variable in class sbrt.shell.mng.stat. MannWhitneyUManager 
 The set of keywords required by this process manager.
   requiredKeywords   - 
Static variable in class sbrt.shell.mng.utilities. IntervalComparisonManager 
 The set of keywords required by this process manager.
   requiredKeywords   - 
Static variable in class sbrt.shell.mng.utilities. VariableParticipationManager 
 The set of keywords required to be present in the  ManagerInput 
 objects supplied to instances of this class.
   requiredKeywords   - 
Static variable in class sbrt.shell.mng.utilities. VectorComparisonManager 
 The set of keywords required by this process manager.
   resetCount()   - 
Method in class sbrt.kernel.util. AbstractProgressMonitor 
 Causes this progress monitor to reset it's internal counter to 0.
   resetCount()   - 
Method in interface sbrt.kernel.util. MutableProgressMonitor 
 Causes this progress monitor to reset its internal counter to 0.
   resetCount()   - 
Method in class sbrt.kernel.util. PercentProgressMonitor 
 Causes this progress monitor to reset it's internal counter to 0.
   restoreColExprConstraint(MathExpr<?>)   - 
Method in class sbrt.kernel.fba.comp. FbaOptComputer 
 Restores the constraint on the provided expression of column variables
 to the value contained in the fluxome.
   restoreColExprConstraints(Collection<? extends MathExpr<?>>)   - 
Method in class sbrt.kernel.fba.comp. FbaOptComputer 
 Restores the constraints for the provided column variable expressions to
 those in the provided fluxome.
   restoreColExprConstraints()   - 
Method in class sbrt.kernel.fba.comp. FbaOptComputer 
 Restores the constraints on all mathematical expressions of column
 variables.
   restoreColumnConstraint(Object)   - 
Method in class sbrt.kernel.fba.comp. FbaOptComputer 
 Restores the constraint for the provided column variable 
 to the value contained in the fluxome.
   restoreColumnConstraints(Collection<?>)   - 
Method in class sbrt.kernel.fba.comp. FbaOptComputer 
 Restores the constraints for the provided column variables to those in
 the provided fluxome.
   restoreColumnConstraints()   - 
Method in class sbrt.kernel.fba.comp. FbaOptComputer 
 Restores the constraints on all column variables to those contained 
 in the fluxome used by this optimization computer.
   restoreConstraints(FluxConstraints, FluxCvComputer)   - 
Static method in class sbrt.kernel.fba.proc. CvFbaOptProcess 
 Restores the provided constraints in the provided constraint-variation 
 computer.
   restoreFluxConstraint(String)   - 
Method in class sbrt.kernel.fba.comp. AbstractFluxIntervalComputer 
 Restores the flux constraint for the reaction with the provided 
 name to the value contained in the fluxome.
   restoreFluxConstraint(String)   - 
Method in class sbrt.kernel.fba.comp. CdHarFluxVectorComputer 
 Restores the flux constraint for the reaction with the provided name to
 the value contained in the fluxome.
   restoreFluxConstraint(String)   - 
Method in class sbrt.kernel.fba.comp. FastMsFluxRangeComputer 
 Restores the flux constraint for the reaction with the provided 
 name to the value contained in the fluxome.
   restoreFluxConstraint(String)   - 
Method in class sbrt.kernel.fba.comp. FbaOptComputer 
 Restores the flux constraint for the reaction with the provided 
 name to the value contained in the fluxome.
   restoreFluxConstraint(String)   - 
Method in interface sbrt.kernel.fba.comp. FluxCvComputer 
 Restores the flux constraint for the reaction with the provided 
 name to the value contained in the fluxome.
   restoreFluxConstraint(String)   - 
Method in class sbrt.kernel.fba.comp. InitialPointComputer 
 Restores the flux constraint for the reaction with the provided 
 name to the value contained in the fluxome.
   restoreFluxConstraint(String)   - 
Method in class sbrt.kernel.fba.comp. RxnDeletionComputer 
 Restores the flux constraint for the reaction with the provided 
 name to the value contained in the fluxome.
   restoreFluxConstraints(Collection<String>)   - 
Method in class sbrt.kernel.fba.comp. AbstractCvComputer 
 Restores the flux constraints for the provided reaction names to the
 values contained in the fluxome.
   restoreFluxConstraints()   - 
Method in class sbrt.kernel.fba.comp. AbstractCvComputer 
 Restores the flux constraints for all reactions in the fluxome to those 
 values contained in the fluxome.
   restoreFluxConstraints(Collection<String>)   - 
Method in class sbrt.kernel.fba.comp. AbstractFluxIntervalComputer 
 Restores the flux constraints for the provided reaction names to the
 values contained in the fluxome.
   restoreFluxConstraints()   - 
Method in class sbrt.kernel.fba.comp. AbstractFluxIntervalComputer 
 Restores the flux constraints for all reactions in the fluxome to those 
 values contained in the fluxome.
   restoreFluxConstraints(Collection<String>)   - 
Method in class sbrt.kernel.fba.comp. FastMsFluxRangeComputer 
 Restores the flux constraints for the provided reaction names to the
 values contained in the fluxome.
   restoreFluxConstraints()   - 
Method in class sbrt.kernel.fba.comp. FastMsFluxRangeComputer 
 Restores the flux constraints for all reactions in the fluxome to those 
 values contained in the fluxome.
   restoreFluxConstraints(Collection<String>)   - 
Method in class sbrt.kernel.fba.comp. FbaOptComputer 
 Restores the constraints for the provided reaction names to those in the
 provided fluxome.
   restoreFluxConstraints()   - 
Method in class sbrt.kernel.fba.comp. FbaOptComputer 
 Restores the flux constraints for all reactions to those contained 
 in the fluxome used by this optimization computer.
   restoreFluxConstraints()   - 
Method in interface sbrt.kernel.fba.comp. FluxCvComputer 
 Restores the flux constraints for all reactions in the fluxome to those 
 values contained in the fluxome.
   restoreFluxConstraints(Collection<String>)   - 
Method in interface sbrt.kernel.fba.comp. FluxCvComputer 
 Restores the flux constraints for the provided reaction names to the
 values contained in the fluxome.
   restoreFluxConstraints(Collection<String>)   - 
Method in class sbrt.kernel.fba.comp. InitialPointComputer 
 Restores the flux constraints for the provided reaction names to the
 values contained in the fluxome.
   restoreFluxConstraints()   - 
Method in class sbrt.kernel.fba.comp. InitialPointComputer 
 Restores the flux constraints for all reactions in the fluxome to those 
 values contained in the fluxome.
   restoreMatrixElements()   - 
Method in class sbrt.kernel.fba.comp. FbaOptComputer 
 Restores the program matrix to the stoichiometry matrix of the fluxome
 used by this computer.
   restoreRowConstraint(Object)   - 
Method in class sbrt.kernel.fba.comp. FbaOptComputer 
 Restores the constraint on the provided row variable.
   restoreRowConstraints(Collection<?>)   - 
Method in class sbrt.kernel.fba.comp. FbaOptComputer 
 Restores the constraints for the provided row variables to those in the
 provided fluxome.
   restoreRowConstraints()   - 
Method in class sbrt.kernel.fba.comp. FbaOptComputer 
 Restores the constraints on all row variables to those contained in 
 the fluxome used by this optimization computer.
   restoreRxnExprConstraint(MathExpr<String>)   - 
Method in class sbrt.kernel.fba.comp. AbstractFluxIntervalComputer 
 Restores the constraint on the provided flux expression to the value
 contained in the fluxome.
   restoreRxnExprConstraint(MathExpr<String>)   - 
Method in class sbrt.kernel.fba.comp. CdHarFluxVectorComputer 
 Restores the constraint on the provided flux expression to the value
 contained in the fluxome.
   restoreRxnExprConstraint(LinearComb<String>)   - 
Method in class sbrt.kernel.fba.comp. CdHarFluxVectorComputer 
 Restores the constraint on the provided flux expression to the value
 contained in the fluxome.
   restoreRxnExprConstraint(MathExpr<String>)   - 
Method in class sbrt.kernel.fba.comp. FastMsFluxRangeComputer 
 Restores the constraint on the provided flux expression to the value
 contained in the fluxome.
   restoreRxnExprConstraint(MathExpr<String>)   - 
Method in class sbrt.kernel.fba.comp. FbaOptComputer 
 Restores the constraint on the provided flux expression to the value
 contained in the fluxome.
   restoreRxnExprConstraint(MathExpr<String>)   - 
Method in interface sbrt.kernel.fba.comp. FluxCvComputer 
 Restores the constraint on the provided flux expression to the value
 contained in the fluxome.
   restoreRxnExprConstraint(MathExpr<String>)   - 
Method in class sbrt.kernel.fba.comp. InitialPointComputer 
 Restores the constraint on the provided flux expression to the value
 contained in the fluxome.
   restoreRxnExprConstraint(MathExpr<String>)   - 
Method in class sbrt.kernel.fba.comp. RxnDeletionComputer 
 Restores the constraint on the provided flux expression to the value
 contained in the fluxome.
   restoreRxnExprConstraints(Collection<? extends MathExpr<String>>)   - 
Method in class sbrt.kernel.fba.comp. AbstractCvComputer 
 Restores the constraints on the provided expressions to the values
 contained in the fluxome.
   restoreRxnExprConstraints(Collection<? extends MathExpr<String>>)   - 
Method in class sbrt.kernel.fba.comp. AbstractFluxIntervalComputer 
 Restores the constraints on the provided expressions to the values
 contained in the fluxome.
   restoreRxnExprConstraints()   - 
Method in class sbrt.kernel.fba.comp. AbstractFluxIntervalComputer 
 Restores the constraints on all flux expressions to the values
 contained in the fluxome.
   restoreRxnExprConstraints()   - 
Method in class sbrt.kernel.fba.comp. CdHarFluxVectorComputer 
 Restores the constraints on all flux expressions to the values contained
 in the fluxome.
   restoreRxnExprConstraints(Collection<? extends MathExpr<String>>)   - 
Method in class sbrt.kernel.fba.comp. FastMsFluxRangeComputer 
 Restores the constraints on the provided expressions to the values
 contained in the fluxome.
   restoreRxnExprConstraints()   - 
Method in class sbrt.kernel.fba.comp. FastMsFluxRangeComputer 
 Restores the constraints on all flux expressions to the values
 contained in the fluxome.
   restoreRxnExprConstraints(Collection<? extends MathExpr<String>>)   - 
Method in class sbrt.kernel.fba.comp. FbaOptComputer 
 Restores the constraints for the provided reaction name expressions to
 those in the provided fluxome.
   restoreRxnExprConstraints()   - 
Method in class sbrt.kernel.fba.comp. FbaOptComputer 
 Restores the constraints on all mathematical expressions of reaction
 names.
   restoreRxnExprConstraints()   - 
Method in interface sbrt.kernel.fba.comp. FluxCvComputer 
 Restores the constraints on all flux expressions to the values
 contained in the fluxome.
   restoreRxnExprConstraints(Collection<? extends MathExpr<String>>)   - 
Method in interface sbrt.kernel.fba.comp. FluxCvComputer 
 Restores the constraints on the provided expressions to the values
 contained in the fluxome.
   restoreRxnExprConstraints(Collection<? extends MathExpr<String>>)   - 
Method in class sbrt.kernel.fba.comp. InitialPointComputer 
 Restores the constraints on the provided expressions to the values
 contained in the fluxome.
   restoreRxnExprConstraints()   - 
Method in class sbrt.kernel.fba.comp. InitialPointComputer 
 Restores the constraints on all flux expressions to the values
 contained in the fluxome.
   restoreRxnExprConstraints()   - 
Method in class sbrt.kernel.fba.comp. RxnDeletionComputer 
 Restores the constraints on all flux expressions to the values
 contained in the fluxome.
   ResultsManager  < T > - Interface in  sbrt.kernel.io  This interace is used to represent a results-manager.   retainAll(Collection<?>)   - 
Method in class sbrt.kernel.util. UnmodifiableCollection 
 See Collection.retainAll(Collection).
   retainAll(Collection<?>)   - 
Method in class sbrt.kernel.util. UnmodifiableList 
 See List.retainAll(Collection).
   retainAll(Collection<?>)   - 
Method in class sbrt.kernel.util. UnmodifiableSet 
 See Set.retainAll(Collection).
   ReverseRxnFluxIntervalComputer   - Class in  sbrt.kernel.fba.comp  This class is used to compute flux intervals while constraining the fluxes of
 reverse reactions to their minimum.   ReverseRxnFluxIntervalComputer(FbaOptComputer, double)   - 
Constructor for class sbrt.kernel.fba.comp. ReverseRxnFluxIntervalComputer 
 Constructs a new flux interval computer.
   ReverseRxnFluxIntervalManager   - Class in  sbrt.shell.mng.fba  This class is used to manage constrained-reverse reaction flux interval experiments.   ReverseRxnFluxIntervalManager()   - 
Constructor for class sbrt.shell.mng.fba. ReverseRxnFluxIntervalManager 
  
   ReversibleRxn   - Class in  sbrt.kernel.fba  This class is used to represent reversible chemical reactions.   ReversibleRxn(LinearComb<String>, LinearComb<String>)   - 
Constructor for class sbrt.kernel.fba. ReversibleRxn 
 Constructs a new reversible reaction from the provided reactants and
 products.
   REVISION   - 
Static variable in class sbrt. Sbrt 
 Indicates the revision number of the Systems Biology Research Tool.
   RevRxnFormatV1   - Class in  sbrt.shell.text.chem  This class is used to format reversible chemical reactions.   RevRxnFormatV1()   - 
Constructor for class sbrt.shell.text.chem. RevRxnFormatV1 
 Constructs a new reversible reaction format.
   RevRxnFormatV2   - Class in  sbrt.shell.text.chem  This class is used to format reversible chemical reactions.   RevRxnFormatV2()   - 
Constructor for class sbrt.shell.text.chem. RevRxnFormatV2 
 Constructs a new reversible reaction format.
   rFlushConsole(Rengine)   - 
Method in class sbrt.ext.R. DummyConsole 
 Does nothing.
   RIGHT_BRACE   - 
Static variable in class sbrt.shell.text. SpecialChars 
 {
   RIGHT_BRACKET   - 
Static variable in class sbrt.shell.text. SpecialChars 
 ]
   RIGHT_PAREN   - 
Static variable in class sbrt.shell.text. SpecialChars 
 )
   rigid()   - 
Method in class sbrt.kernel.fba.proc. FluxPlasticityProcess.Results 
 Returns the number of rigid fluxes.
   RKendall  < V > - Class in  sbrt.ext.R.stat  This class computes Kendall tau values using R.   RKendall()   - 
Constructor for class sbrt.ext.R.stat. RKendall 
  
   rLoadHistory(Rengine, String)   - 
Method in class sbrt.ext.R. DummyConsole 
 Does nothing.
   RofoofSource   - Class in  sbrt.shell.io.fba  This class is used to provide a source of row-oriented
 FBA optimization output files.   RofoofSource(Iterator<String>, FileFormat, Set<String>, FbaOptFileLineFormat)   - 
Constructor for class sbrt.shell.io.fba. RofoofSource 
 Constructs a new source of row-oriented
 FBA optimization output files.
   RofoofSource(Iterator<String>, String, FileFormat, Set<String>, FbaOptFileLineFormat)   - 
Constructor for class sbrt.shell.io.fba. RofoofSource 
 Constructs a new source of row-oriented
 FBA optimization output files.
   RomiFile  < K , V > - Class in  sbrt.shell.io  This class is used to read row-oriented map input files.   RomiFile(String, FileFormat, Set<? extends K>, MapFormat<K, V>)   - 
Constructor for class sbrt.shell.io. RomiFile 
 Constructs a new row-oriented map input file from the provided arguments.
   RomiFile(String, FileFormat, MapFormat<K, V>)   - 
Constructor for class sbrt.shell.io. RomiFile 
 Constructs a new row-oriented map input file from the provided arguments.
   RomiFile(String, MapFormat<K, V>)   - 
Constructor for class sbrt.shell.io. RomiFile 
 Constructs a new row-oriented map input file from the provided arguments.
   RomiFile(String, Set<? extends K>, MapFormat<K, V>)   - 
Constructor for class sbrt.shell.io. RomiFile 
 Constructs a new row-oriented map input file from the provided arguments.
   RomiFileSource  < K , V > - Class in  sbrt.shell.io  This class is used to provided a source of row-oriented map input files.   RomiFileSource(Iterator<String>, FileFormat, Set<? extends K>, MapFormat<K, V>)   - 
Constructor for class sbrt.shell.io. RomiFileSource 
 Constructs a new source of row-oriented map input files.
   RomiFileSource(Iterator<String>, FileFormat, MapFormat<K, V>)   - 
Constructor for class sbrt.shell.io. RomiFileSource 
 Constructs a new source of row-oriented map input files.
   RomiFileSource(Iterator<String>, MapFormat<K, V>)   - 
Constructor for class sbrt.shell.io. RomiFileSource 
 Constructs a new source of row-oriented map input files.
   RomoFile  < K , V > - Class in  sbrt.shell.io  This class is used to write maps to text files in a 
 row-oriented way.   RomoFile(String, FileFormat, Set<? extends K>, MapFormat<K, V>)   - 
Constructor for class sbrt.shell.io. RomoFile 
 Constructs a new row-oriented map output file 
 from the provided arguments.
   RomoFile(String, FileFormat, String, MapFormat<K, V>)   - 
Constructor for class sbrt.shell.io. RomoFile 
 Constructs a new row-oriented vector output file 
 from the provided arguments.
   RomoFile(String, FileFormat, MapFormat<K, V>)   - 
Constructor for class sbrt.shell.io. RomoFile 
 Constructs a new row-oriented vector output file from the
 provided arguments.
   RomoFile(String, MapFormat<K, V>)   - 
Constructor for class sbrt.shell.io. RomoFile 
 Constructs a new plain text, row-oriented vector output 
 file from the provided arguments.
   RomoFileSource  < K , V > - Class in  sbrt.shell.io  This class is used to provided a source of row-oriented map output files.   RomoFileSource(Iterator<String>, FileFormat, Set<? extends K>, MapFormat<K, V>)   - 
Constructor for class sbrt.shell.io. RomoFileSource 
 Constructs a new source of row-oriented map output files.
   RomoFileSource(Iterator<String>, FileFormat, String, MapFormat<K, V>)   - 
Constructor for class sbrt.shell.io. RomoFileSource 
 Constructs a new source of row-oriented map output files.
   RomoFileSource(Iterator<String>, FileFormat, MapFormat<K, V>)   - 
Constructor for class sbrt.shell.io. RomoFileSource 
 Constructs a new source of row-oriented map output files.
   RowOrFbaOptOutputFile   - Class in  sbrt.shell.io.fba  This class is used to write row-oriented output files 
 containing FBA optimization data.   RowOrFbaOptOutputFile(String, FileFormat, Set<String>, FbaOptFileLineFormat)   - 
Constructor for class sbrt.shell.io.fba. RowOrFbaOptOutputFile 
 Constructs a new row-oriented FBA optimization output file.
   RowOrFbaOptOutputFile(String, FileFormat, FbaOptFileLineFormat)   - 
Constructor for class sbrt.shell.io.fba. RowOrFbaOptOutputFile 
 Constructs a new row-oriented FBA optimization output file.
   RowOrTreeMatrix  < R  extends java.lang.Comparable<? super  R >, C  extends java.lang.Comparable<? super  C >> - Class in  sbrt.kernel.math.algebra  This class is a  java.util.TreeMap   based implementation of   SmdMatrix  .   RowOrTreeMatrix()   - 
Constructor for class sbrt.kernel.math.algebra. RowOrTreeMatrix 
 Constructs an emtpy  RowOrTreeMatrix .
   rows()   - 
Method in class sbrt.kernel.math.algebra. HashMatrix 
 Returns the number of rows in this matrix.
   rows()   - 
Method in interface sbrt.kernel.math.algebra. Matrix 
 Returns the number of rows in this matrix.
   rows()   - 
Method in class sbrt.kernel.math.algebra. MatrixWrap 
 Returns the number of rows in this matrix.
   rows()   - 
Method in class sbrt.kernel.math.algebra. RowOrTreeMatrix 
 Returns the number of rows in this matrix.
   rows()   - 
Method in class sbrt.kernel.math.algebra. TreeMatrix 
 Returns the number of rows in this matrix.
   RPearson  < V > - Class in  sbrt.ext.R.stat  This class computes Pearson r values using R.   RPearson()   - 
Constructor for class sbrt.ext.R.stat. RPearson 
  
   rReadConsole(Rengine, String, int)   - 
Method in class sbrt.ext.R. DummyConsole 
 Does nothing.
   rSaveHistory(Rengine, String)   - 
Method in class sbrt.ext.R. DummyConsole 
 Does nothing.
   rShowMessage(Rengine, String)   - 
Method in class sbrt.ext.R. DummyConsole 
 Does nothing.
   RSpearman  < V > - Class in  sbrt.ext.R.stat  This class computes Spearman rho values using R.   RSpearman()   - 
Constructor for class sbrt.ext.R.stat. RSpearman 
  
   run()   - 
Method in class sbrt.kernel.fba.comp. CdHarFluxVectorComputer 
 Computes the next random flux vector from within the interior of the
 convex polytope defined by the current set of constraints.
   run()   - 
Method in class sbrt.kernel.fba.comp. InitialPointComputer 
 Computes an initial point, or flux vector, in the flux space of the
 fluxome used by this computer.
   run(ResultsManager<FbaOptComputer.Results>)   - 
Method in class sbrt.kernel.fba.proc. CatalystDeletionProcess 
 Runs this process and adds the generated results to the provided results
 manager via its   ResultsManager.addData(Object)   method.
   run(ResultsManager<CdHarFluxVectorComputer.Results>)   - 
Method in class sbrt.kernel.fba.proc. CdHarFluxVectorProcess 
 Generates the specified number of random flux vectors and adds each to
 the provided results manager.
   run(ResultsManager<FbaOptComputer.Results>)   - 
Method in class sbrt.kernel.fba.proc. CvCatalystDeletionProcess 
 Runs this process and adds the generated results to the provided results
 manager via its   ResultsManager.addData(Object)   method.
   run(ResultsManager<FbaOptComputer.Results>)   - 
Method in class sbrt.kernel.fba.proc. CvFbaOptimizationExperiment 
 Runs this process and adds the generated
 results to the provided results manager via
 its   ResultsManager.addData(Object)   
 method.
   run(ResultsManager<Map<String, Interval>>)   - 
Method in class sbrt.kernel.fba.proc. CvFluxIntervalProcess 
 Runs this process and adds the generated results to the provided results
 manager via its   ResultsManager.addData(Object)   method.
   run(ResultsManager<FbaOptComputer.Results>)   - 
Method in class sbrt.kernel.fba.proc. CvObjectiveFunctionProcess 
 Runs this process and adds the generated results to the provided results
 manager via its   ResultsManager.addData(Object)   method.
   run(ResultsManager<FbaOptComputer.Results>)   - 
Method in class sbrt.kernel.fba.proc. CvRxnDeletionProcess 
 Runs this process and adds the generated results to the provided results
 manager via its   ResultsManager.addData(Object)   method.
   run(ResultsManager<DoubleMatrix<?, String>>)   - 
Method in class sbrt.kernel.fba.proc. CycleIdProcess 
 Runs this process and adds the generated results to the provided results
 manager via its   ResultsManager.addData(Object)   method.
   run(ResultsManager<DoubleMatrix<?, String>>)   - 
Method in class sbrt.kernel.fba.proc. ExtremeCurrentProcess 
 Runs this process and adds the generated
 results to the provided results manager via
 its   ResultsManager.addData(Object)   
 method.
   run(ResultsManager<FbaOptComputer.Results>)   - 
Method in class sbrt.kernel.fba.proc. FbaOptimizationProcess 
 Computes the optimal value of the current objective function and adds the
 results to the provided results manager via its
   ResultsManager.addData(Object)   method.
   run(ResultsManager<LinearSystemSolution<String>>)   - 
Method in class sbrt.kernel.fba.proc. FbaSolverProcess 
 Solves Sv = 0 and adds the result to the provided manager.
   run(ResultsManager<FluxActivityProcess.Results>)   - 
Method in class sbrt.kernel.fba.proc. FluxActivityProcess 
 Iterates thru the flux vector source set via 
  setFluxVectorSource(Iterable) , counts the number of active and
 inactive fluxes in each vector, and adds the results of each to the
 provided results manager.
   run(ResultsManager<Map<String, Set<LinearComb<String>>>>)   - 
Method in class sbrt.kernel.fba.proc. FluxCapProcess 
 Computes the flux caps and adds the result to the provided manager.
   run(ResultsManager<Map<String, Interval>>)   - 
Method in class sbrt.kernel.fba.proc. FluxIntervalProcess 
 Computes the flux ranges of the reactions with the current names and
 adds them to the provided results manager.
   run(ResultsManager<T>)   - 
Method in class sbrt.kernel.fba.proc. FluxomeReducerProcess 
 Runs this process and adds the generated results to the provided results
 manager via its   ResultsManager.addData(Object)   method.
   run(ResultsManager<FluxPlasticityProcess.Results>)   - 
Method in class sbrt.kernel.fba.proc. FluxPlasticityProcess 
 Iterates thru the flux range source set via 
  setFluxRangeSource(Iterable) , computes the flux plasticity
 measures, and adds the results of each to the
 provided results manager.
   run(ResultsManager<InitialPointComputer.Results>)   - 
Method in class sbrt.kernel.fba.proc. InitialPointGenerator 
 Generates an initial point and adds it to the provided results manager.
   run(ResultsManager<NetworkInfoProcess.Results>)   - 
Method in class sbrt.kernel.fba.proc. NetworkInfoProcess 
 Adds the information gathered about the fluxome to the provided
 results manager.
   run(ResultsManager<FbaOptComputer.Results>)   - 
Method in class sbrt.kernel.fba.proc. ObjectiveFunctionAnalysis 
 Runs this process and adds the generated
 results to the provided results manager via
 its   ResultsManager.addData(Object)   
 method.
   run(ResultsManager<Map<String, Interval>>)   - 
Method in class sbrt.kernel.fba.proc. RandomConstraintsGeneratorV1 
 Generates sets of random flux constraints and adds them to the provided
 results manager.
   run(ResultsManager<Map<String, Interval>>)   - 
Method in class sbrt.kernel.fba.proc. RandomEnvGeneratorV1 
 Generates a random environment and adds it to the provided
 results manager.
   run(ResultsManager<LinearComb<String>>)   - 
Method in class sbrt.kernel.fba.proc. RandomObjectiveGenerator 
 Generates random objective functions and adds them to the provided
 results manager.
   run(ResultsManager<FbaOptComputer.Results>)   - 
Method in class sbrt.kernel.fba.proc. RxnDeletionProcess 
 Runs this process and adds the generated
 results to the provided results manager via
 its   ResultsManager.addData(Object)   
 method.
   run(ResultsManager<FbaOptComputer.Results>)   - 
Method in class sbrt.kernel.fba.proc. StoichVariationProcess 
 Runs this process and adds the generated results to the provided results
 manager via its   ResultsManager.addData(Object)   method.
   run(ResultsManager<CdHarFluxVectorComputer.Results>)   - 
Method in class sbrt.kernel.fba.proc. SvCdHarFluxVectorProcess 
 Runs this process and adds the generated results to the provided results
 manager via its   ResultsManager.addData(Object)   method.
   run(ResultsManager<InitialPointComputer.Results>)   - 
Method in class sbrt.kernel.fba.proc. SvInitialPointProcess 
 Runs this process and adds the generated results to the provided results
 manager via its   ResultsManager.addData(Object)   method.
   run(ResultsManager<T>)   - 
Method in interface sbrt.kernel. KernelProcess 
 Runs this process and adds the generated
 results to the provided results manager via
 its   ResultsManager.addData(Object)   
 method.
   run(ResultsManager<LinearSystemSolution<V>>)   - 
Method in class sbrt.kernel.math.algebra.proc. LinearSolverProcess 
 Solves the system of linear equations and adds the solution 
 to the provided results manager.
   run(ResultsManager<Map<K, V>>)   - 
Method in class sbrt.kernel.math.algebra.proc. VectorIntersection 
 and adds the results to the provided results manager.
   run(ResultsManager<Set<Set<E>>>)   - 
Method in class sbrt.kernel.math.comb.proc. SingleElementUnionProc 
 Computes the single-element unions of the provided collection of sets,
 and adds the results to the provided results manager.
   run(ResultsManager<Set<Set<E>>>)   - 
Method in class sbrt.kernel.math.comb.proc. StrictSingleElementUnionProc 
 Computes the strict single-element unions of the provided collection of sets,
 and adds the results to the provided results manager.
   run(Map<? extends V, Double>)   - 
Method in class sbrt.kernel.math.geom.comp. CdHarComputer 
 Computes and returns a random interior point of the convex polytope
 starting from the provided point.
   run(ResultsManager<CdHarComputer.Results>)   - 
Method in class sbrt.kernel.math.geom.proc. CdHarProcess 
 Adds random points to the provided results manager.
   run(ResultsManager<Path<N>>)   - 
Method in class sbrt.kernel.math.graph_theory.proc. CycleIdProcess 
 Adds to the provided results manager each identified cycle in the current
 graph.
   run(ResultsManager<Path<N>>)   - 
Method in class sbrt.kernel.math.graph_theory.proc. PathIdProcess 
 Adds to the provided results manager each computed path in the current
 graph.
   run(ResultsManager<T>)   - 
Method in class sbrt.kernel.math.stat.proc. CorrelationAnalysis 
 Computes the correlation statistic between the x and y values,
 and adds the results to the provided results manager.
   run(ResultsManager<T>)   - 
Method in class sbrt.kernel.math.stat.proc. MultipleCorrelationAnalysis 
 Computes the correlation statistic between the x and y values, and adds
 the results to the provided results manager.
   run(ResultsManager<MannWhitneyResults>)   - 
Method in class sbrt.kernel.math.stat.proc. MwUAnalysis 
 Computes the Mann-Whitney U statistic of the samples A and B,
 and adds the results to the provided results manager.
   run(ResultsManager<Set<IntervalComparer.Diff<V>>>)   - 
Method in class sbrt.kernel.utilities.proc. IntervalComparison 
 Adds to the provided results manager the variables (keys) whose intervals
 (values) are unequal within the provided tolerance.
   run(ResultsManager<Map<V, List<E>>>)   - 
Method in class sbrt.kernel.utilities.proc. VariableParticipation 
 Adds to the provided results manager a map of variables mapped to a
 list of mathematical expressions in which they participate.
   run(ResultsManager<Map<V, Double>>)   - 
Method in class sbrt.kernel.utilities.proc. VectorComparison 
 Adds to the provided results manager the variables (keys) whose intervals
 (values) are unequal within the provided tolerance.
   run(String, PrintWriter)   - 
Method in class sbrt. ProcessRunner 
 Executes the process specified in the indicated process file.
   run(String, OutputStream)   - 
Method in class sbrt. ProcessRunner 
 Executes the process specified in the indicated process file.
   run(String, PrintStream)   - 
Method in class sbrt. ProcessRunner 
 Executes the process specified in the indicated process file.
   run(ManagerInput<String, String>, OutputStream)   - 
Method in class sbrt. ProcessRunner 
 Executes the process specified in the provided process manager input.
   run(ManagerInput<String, String>, PrintStream)   - 
Method in class sbrt. ProcessRunner 
 Executes the process specified in the provided process manager input.
   run(ManagerInput<String, String>, PrintWriter)   - 
Method in class sbrt. ProcessRunner 
 Executes the process specified in the provided process manager input.
   runProcess(MonitoredProcess<T>, ResultsManager<T>, PrintWriter)   - 
Static method in class sbrt.shell.mng. AbstractProcessManager 
 Runs the provided process using the provided results manager and prints
 descriptive progress messages to the provided print writer.
   rWriteConsole(Rengine, String, int)   - 
Method in class sbrt.ext.R. DummyConsole 
 Does nothing.
   RXN_CATALYST_FILE   - 
Static variable in class sbrt.shell.mng.fba. FbaManager 
 The keyword used to denote the name of a file containing reaction-catalyst
 associations.
   RXN_DELETION_FILE   - 
Static variable in class sbrt.shell.mng.fba. RxnDeletionManager 
 The keyword used to denote the name of a file containing sets of
 names of reactions whose effect upon deletion will be computed.
   RXN_FILE   - 
Static variable in class sbrt.shell.mng.fba. FbaManager 
 The keyword used to denote an FBA reaction file.
   RXN_FILE_NAME   - 
Static variable in class sbrt.shell.mng.fba. BiggSbmlReaderManager 
 Used to denote the name of the file to which chemical reactions should be written.
   RXN_FILE_NAME   - 
Static variable in class sbrt.shell.mng.fba. BiggSbmlTranslaterManager 
 Used to denote the name of the file to chemical reactions should be written.
   RXN_FILE_NAME   - 
Static variable in class sbrt.shell.mng.fba. PalssonSbmlReaderManager 
 Used to denote the name of the file to which chemical reactions should be written.
   RXN_FILE_NAME   - 
Static variable in class sbrt.shell.mng.fba. PalssonSbmlTranslaterManager 
 Used to denote the name of the file to chemical reactions should be written.
   RXN_NAME_FILE   - 
Static variable in class sbrt.shell.mng.fba. RxnRemovalManager 
 The keyword used to denote the name of the file that contains the
 names of the reactions to be removed.
   RXN_NAME_FILE_NAME   - 
Static variable in class sbrt.shell.mng.fba. NetworkInfoManager 
 The keyword used to denote the name of the file to which the names of
 reactions of a fluxome will be written.
   RXN_NODE_NAME   - 
Static variable in class sbrt.shell.text.fba. BiggRxnNodeParser 
 The name of the nodes parsed by this class ('reaction').
   RXN_NODE_NAME   - 
Static variable in class sbrt.shell.text.fba. PalssonRxnNodeParser 
 The name of the nodes parsed by this class ('reaction').
   RXN_OUTPUT_FILE_NAME   - 
Static variable in class sbrt.shell.mng.fba. NetworkInfoManager 
 The keyword used to denote the name of the file to which a fluxome will
 be written.
   RxnDeletionComputer   - Class in  sbrt.kernel.fba.comp  This class is used to compute the effect of deleting reactions in a
 fluxome.   RxnDeletionComputer(FbaOptComputer)   - 
Constructor for class sbrt.kernel.fba.comp. RxnDeletionComputer 
 Constructs a new reaction deletion computer.
   RxnDeletionManager   - Class in  sbrt.shell.mng.fba  This class is used to manage FBA reaction deletion experiments.   RxnDeletionManager()   - 
Constructor for class sbrt.shell.mng.fba. RxnDeletionManager 
  
   RxnDeletionProcess   - Class in  sbrt.kernel.fba.proc  This class is used to perform reaction deletion experiments.   RxnDeletionProcess(RxnDeletionComputer)   - 
Constructor for class sbrt.kernel.fba.proc. RxnDeletionProcess 
 Constructs a new reaction deletion experiment.
   RxnNameExprFormat  < E  extends  MathExpr < S >, S > - Interface in  sbrt.shell.text.fba  This interface is used to represent formats for mathematical
 expressions of reaction names.   RxnNameExprFormatV1   - Class in  sbrt.shell.text.fba  This class is used to format linear combinations of reaction names.   RxnNameExprFormatV1(Fluxome)   - 
Constructor for class sbrt.shell.text.fba. RxnNameExprFormatV1 
 Constructs a new reaction name expression format
 using the provided fluxome.
   RxnNameFormat  < T > - Interface in  sbrt.shell.text.chem  This interface is used to represent formats for the
 names of chemical reactions.   RxnNameFormatV1   - Class in  sbrt.shell.text.chem  This class is a concrete implementation of   RxnNameFormat  .   RxnNameFormatV1()   - 
Constructor for class sbrt.shell.text.chem. RxnNameFormatV1 
  
   RxnNameListFormat   - Interface in  sbrt.shell.text.fba  This interface is used to represent the formats of collections 
 of reaction names.   RxnNameListFormatV1   - Class in  sbrt.shell.text.fba  This class is a concrete implemenation of   RxnNameListFormat  .   RxnNameListFormatV1(Fluxome)   - 
Constructor for class sbrt.shell.text.fba. RxnNameListFormatV1 
 Constructs a new reaction name list format
 using the provided fluxome.
   RxnNameOrExprFormat   - Interface in  sbrt.shell.text.fba  This interface is used to represent formats for reaction
 names and mathematical expressions of reaction names.   RxnNameOrExprFormatV1   - Class in  sbrt.shell.text.fba  This class is a concrete implemenation of   RxnNameOrExprFormat  .   RxnNameOrExprFormatV1(Fluxome)   - 
Constructor for class sbrt.shell.text.fba. RxnNameOrExprFormatV1 
 Constructs a new reaction name and expression format.
   RxnNameSetFormat   - Interface in  sbrt.shell.text.fba  This interface is used to represent the formats of collections 
 of reaction names.   RxnNameSetFormatV1   - Class in  sbrt.shell.text.fba  This class is a concrete implemenation of   RxnNameSetFormat  .   RxnNameSetFormatV1(Fluxome)   - 
Constructor for class sbrt.shell.text.fba. RxnNameSetFormatV1 
 Constructs a new reaction name collection format
 using the provided fluxome.
   RxnNameVerifier   - Class in  sbrt.shell.text.fba  This class is used to ensure reaction names exist in a 
 given  Fluxome .   RxnNameVerifier(Fluxome)   - 
Constructor for class sbrt.shell.text.fba. RxnNameVerifier 
 Constructs a new reaction name checker for the 
 provided fluxome.
   RxnNamExprSetInputFile  < E  extends  MathExpr < S >, S > - Class in  sbrt.shell.io.fba  This class is used to read files containing a set of reaction name
 expressions.   RxnNamExprSetInputFile(String, RxnNameExprFormat<E, S>)   - 
Constructor for class sbrt.shell.io.fba. RxnNamExprSetInputFile 
 Constructs a new reaction name expression set input file.
   RxnRemovalManager   - Class in  sbrt.shell.mng.fba  This class is used to manage the removal of reactions from stoichiometric networks.   RxnRemovalManager()   - 
Constructor for class sbrt.shell.mng.fba. RxnRemovalManager 
  
   rxns()   - 
Method in interface sbrt.kernel.fba. Fluxome 
 Returns the total number of chemical reactions contained
 in this fluxome.
   rxns()   - 
Method in class sbrt.kernel.fba. IrrevRxnFluxomeV1 
 Returns the total number of chemical reactions contained
 in this fluxome.
 
 


 
   
  
 
 
 
   
 
   
          Overview     
        Package   
        Class   
        Use   
          Tree     
          Deprecated     
       Index    
          Help     
   
 
 
  
 
 
 

 
  
   PREV LETTER   
   NEXT LETTER    
  
    FRAMES     
   NO FRAMES     
  
    All Classes  ');
  }
  //-->
 
 
    All Classes  
 


  
 
 
  
 

 A   B   C   D   E   F   G   H   I   J   K   L   M   N   O   P   Q   R   S   T   U   V   W   X   Y   Z
